# Are parents’ motivations to exercise and intention to engage in regular family-based activity associated with both adult and child physical activity?

**DOI:** 10.1136/bmjsem-2016-000137

**Published:** 2017-02-12

**Authors:** Emma Solomon-Moore, Simon J Sebire, Janice L Thompson, Jesmond Zahra, Debbie A Lawlor, Russ Jago

**Affiliations:** 1 Centre for Exercise, Nutrition and Health Sciences, School for Policy Studies, University of Bristol, Bristol, UK; 2 School of Sport, Exercise and Rehabilitation Sciences, University of Birmingham, Birmingham, UK; 3 School for Social and Community Medicine, University of Bristol, Bristol, UK; 4 MRC Integrative Epidemiology Unit, University of Bristol, Bristol, UK

**Keywords:** Physical activity, family, parents, motivations, intentions

## Abstract

**Background/aim:**

To examine the associations between parents’ motivation to exercise and intention to engage in family-based activity with their own and their child’s physical activity.

**Methods:**

Cross-sectional data from 1067 parent–child pairs (76.1% mother–child); children were aged 5–6 years. Parents reported their exercise motivation (ie, intrinsic motivation, identified regulation, introjected regulation, external regulation and amotivation) as described in self-determination theory and their intention to engage in family-based activity. Parents’ and children’s mean minutes of moderate-to-vigorous-intensity physical activity (MVPA) and mean counts per minute were derived from ActiGraph accelerometers worn for 3 to 5 days (including a mixture of weekdays and weekend days). Multivariable linear regression models, adjusted for parent sex, number of children, indices of multiple deprivation and clustering of children in schools were used to examine associations (total of 24 associations tested).

**Results:**

In fully adjusted models, each unit increase in identified regulation was associated with a 6.08 (95% CI 3.27 to 8.89, p<0.001) min-per-day increase in parents’ MVPA. Parents’ external regulation was associated with children performing 2.93 (95% CI −5.83 to −0.03, p=0.05) fewer minutes of MVPA per day and a 29.3 (95% CI −53.8 to −4.7, p=0.02) accelerometer count-per-minute reduction. There was no evidence of association for the other 21 associations tested.

**Conclusions:**

Future family-based physical activity interventions may benefit from helping parents identify personal value in exercise while avoiding the use of external control or coercion to motivate behaviour.

**What are the new findings?**

Parents of young children who accept exercise as personally important or valuable to them engage in six more minutes of physical activity per day.
Young children whose parents exercise because of external demands or possible reward engage in almost three less minutes of physical activity per day.
Future family-based physical activity interventions should focus on increasing parents’ autonomous motivation to exercise and avoid using external controls or coercion to motivate behaviour.

## Introduction

Physical inactivity is estimated to cause 6% of deaths globally, making it the fourth leading risk factor for global mortality.^1^ In England, 67% of men and 55% of women self-reported meeting the recommended guidelines of at least 150 min of moderate-intensity or 75 min of vigorous-intensity activity per week.^2^ However, when physical activity (PA) was measured objectively using accelerometers, only 6% of men and 4% of women met the guidelines.^3^ For children aged 5–15 years, only 33% of boys and 21% of girls met the recommended guidelines (≥1 hour per day of moderate-intensity PA).^3^

### Families and PA

Due to the low prevalence of sufficient PA among the population, targeting families may be an important way to increase PA among children and their parents. However, the majority of research concerning the psychosocial determinants of PA has focused on the general adult population,^4^ relying on the assumption that the factors that affect PA are the same for parents and non-parents. Emerging evidence, however, suggests otherwise. The onset of parenthood has been associated with a decline in PA,^5 6^ and parents of young children report having fewer opportunities for PA because they have new responsibilities and time commitments,^7–10^ and their priorities have shifted from themselves to their child.^11^

The best available data suggest that the association between parent and child PA is weak.^12 13^

Parents are, however, an important influence on their children’s PA^14–16^ through the extent to which a parent facilitates PA for their child and parental attitudes towards PA.^15^ Collectively, the research suggests that parents do not have to be active themselves to influence their child’s PA,^17^ but that more research is needed to understand how parents influence their children’s PA.

### Exercise motivation

Motivation to exercise is central to understanding adults’ PA.^18^ Self-determination theory (SDT) is a conceptual framework in which motivation for a given behaviour (eg, PA) is broadly classified as either autonomous or controlled and that these characteristics can either facilitate or hinder behavioural performance and persistence.^19^ Autonomous types of motivation comprise intrinsic motivation (ie, doing a behaviour for its inherent satisfaction/enjoyment), integrated regulation (ie, where performing a behaviour is aligned with one’s identity) and identified regulation (ie, consciously valuing a behaviour).^20^ In contrast, controlled motivation represents less self-determined reasons for behaviour, and comprises introjected regulation (ie, doing something in order to protect one’s ego or to avoid guilt) and external regulation (ie, performing behaviours to satisfy external demands or obtain rewards). While autonomous and controlled motivation vary in their self-determination, amotivation is the lack of motivation to perform a certain behaviour.^20^ Good evidence has been demonstrated for the value of SDT in understanding and promoting exercise behaviour.^21^ Specifically, adults’ autonomous motivation has been found to be positively associated with objectively assessed PA, whereas controlled motivation has not.^22 23^ However, a systematic review identified several studies in which introjected regulation was positively associated with PA,^21^ highlighting the complex nature of the motivation–behaviour relationship.^24–26^ When these studies were examined closely, the strength of association for introjected regulation was lower compared with autonomous types of motivation.^21 24 26^

Parents of young children arguably experience more challenges to converting PA motivation into behaviour than non-parents because of the greater demands on their time and PA being less of a priority. It is important to understand what types of motivation are associated with PA in parents, as opposed to adults in general, and whether autonomous motivation is enough to bridge the motivation–PA gap for busy parents. It is also possible that the quality of parents’ exercise motivation (eg, autonomous vs controlled) could influence their child’s PA. For example, if parents’ motivations are primarily controlled and they feel PA is something that they ‘have to’ rather than ‘want to’ do, when faced with the demands of parenting a young child they may not: (1) authentically engage in PA themselves or (2) have strong intentions to promote PA with their child. A cross-sectional study of parents with young children found parents’ self-determined motivation was indirectly associated with their intention to be active via their attitudes.^27^ However, the motivation variable only represented identified regulation, PA was self-reported, child PA was not measured, and while parents’ intentions were measured, their intention to be active with their child was not. Additionally, previous work has integrated the SDT motivation constructs with the concept of intention.^27 28^ In undergraduate students, autonomous motivation was positively associated with intention, which was in turn associated with exercise behaviour, whereas the associations between controlled motivation, intention and behaviour were relatively modest in comparison, albeit still positive.^29^ To date, no research has explored the association between parents’ autonomous and controlled motivation, their intention to engage in family-based activity and their own and their child’s objectively assessed PA. Further, no research has examined whether intention mediates the motivation–behaviour association. Thus, this study will advance SDT-based research by improving our understanding of the association between motivation and PA within the context of parenthood.

The aim of this research was to examine the associations between parents’ motivation to exercise and their own and their young child’s objectively assessed PA, and whether these associations were mediated by parents’ intention to engage in regular family-based activity. It is hypothesised that parents’ autonomous motivation regulations and intention to engage in family-based activity will be positively associated with PA for both parents and children, while parents’ controlled motivation regulations will be negatively associated with PA.

## Methods

### Sampling

**Figure 1:**
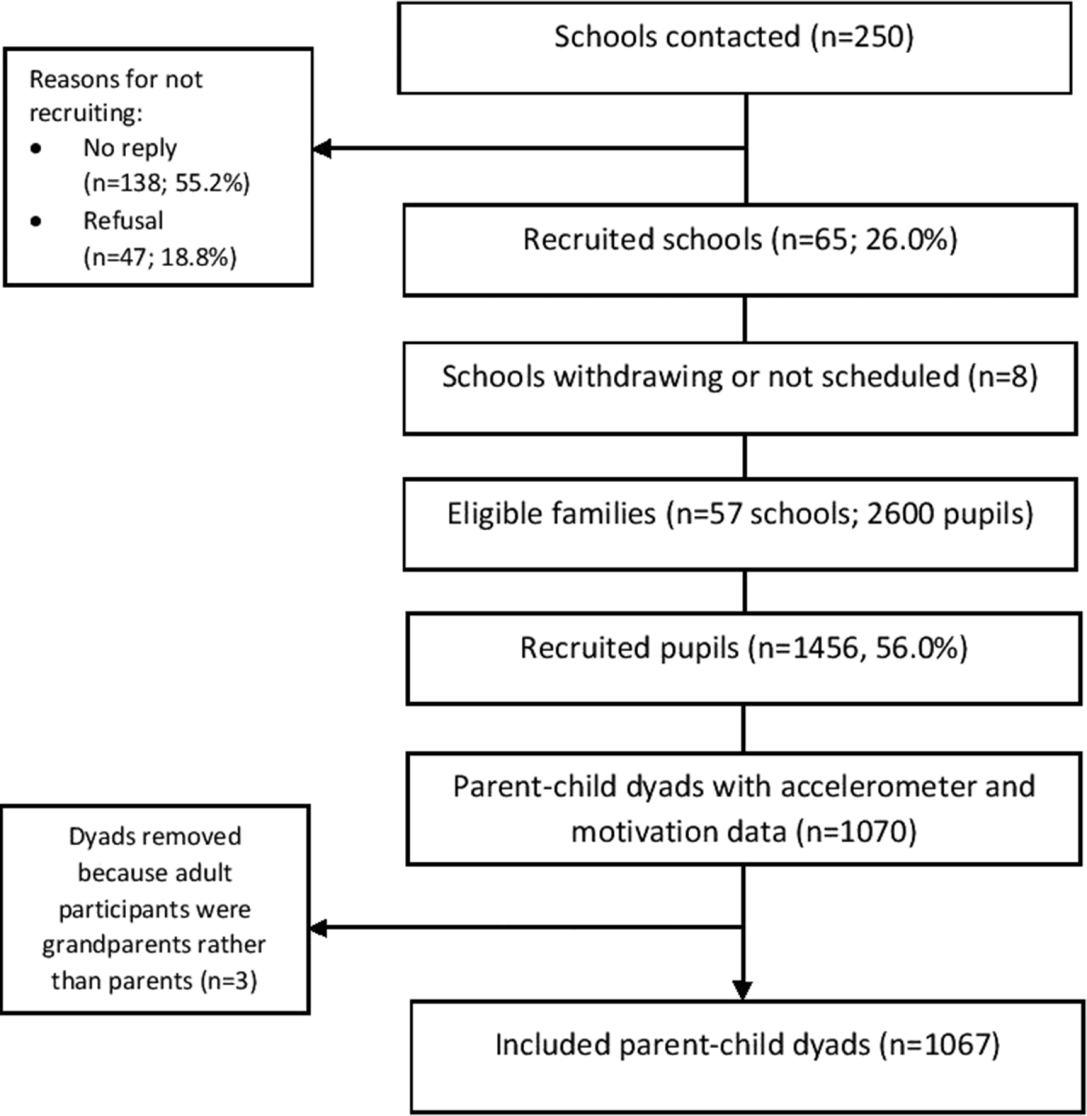
Study flow of participants.

The current analyses used data from a cross-sectional study (B-ProAct1v) carried out at the University of Bristol. The study aimed to identify factors associated with young children’s (5–6 years) and parents’ PA and screen viewing, with a specific focus on the influence of parents on child PA and screen-viewing behaviours. Study design, participant recruitment and data collection methods are described in greater detail elsewhere,^12^ and several papers have been published from the data.^12 30–34^ Briefly, in 2012–2013, data were collected from 57 primary schools in the greater Bristol area, from 250 schools approached (22.8%). Written informed consent was obtained from parents for both the parents’ and children’s participation.^35^ Ethical approval was granted by the School for Policy Studies research ethics committee at the University of Bristol. In total, 1456 child–parent dyads consented to take part. Child–parent dyads of 1267 wore and returned an accelerometer and were included in the final dataset. For the current study, we were interested in parents’ exercise motivations, therefore, results herein are based on data for the 1067 child–parent dyads that provided accelerometer data and completed at least part of the motivation measures. Figure 1 shows the study flow of participants.

### Data collection

Parents were asked to complete a questionnaire about family characteristics, personal demographics, exercise motivations and intention to engage in regular family-based PA. Parents self-reported their sex, height and weight to enable the calculation of body mass index (BMI=kg/m^2^). Indices of Multiple Deprivation (IMD) scores, based upon the English Indices of Deprivation (http://data.gov.uk/dataset/index-of-multiple-deprivation), were assigned to each dyad based on their self-reported home postcode, where higher IMD scores indicate a greater level of local deprivation.

### Physical activity

Children and parents wore an ActiGraph GT3X accelerometer on their waist for 5 days, 3 weekdays and 2 weekend days. ActiGraph GT3X accelerometers have been demonstrated to have good validity for measuring PA in free-living conditions,^36 37^ and waist-worn accelerometers have been demonstrated to outperform wrist-worn accelerometers during laboratory-based step counts.^38^ Parents and children were included in the primary analyses if they provided at least 3 days of valid data (including at least 1 weekend day) to conform with the method used in previous studies.^12^ A valid day was defined as ≥500 min of data, after excluding intervals of ≥60 min of zero counts allowing up to 2 min of interruptions.^39^ Uniaxial data were processed using Kinesoft software (V.3.3.75; Kinesoft, Saskatchewan, Canada). Minutes spent in moderate-to-vigorous-intensity physical activity (MVPA) were derived using population-specific cut points for children and adults.^40 41^ Mean accelerometer counts per minute (CPM) were calculated to provide an indication of volume of PA.

### Motivation and intention

Parents’ motivation to exercise was measured using the 19-item Behavioural Regulation in Exercise Questionnaire (BREQ-2).^42^ The BREQ-2 assesses five forms of exercise motivation regulations: intrinsic, identified, introjected, external and amotivation. Participants rated each item on a 5-point Likert scale ranging from 0 (*not true for me*) to 4 (*very true for me*). In the current study, the internal consistency of the BREQ-2 subscales was: intrinsic (α=0.92), identified (α=0.83), introjected (α=0.75), external (α=0.70) and amotivation (α=0.76).

Intention to regularly engage in family-based PA was measured using a single-item scale adapted from previous research.^43 44^ Participants responded to the stem ‘Thinking about the upcoming month, indicate how many times per week you are intending to engage in regular family-based physical activity’ with an open answer. The range of acceptable scores was 0 to ≥7 (ie, eight categories).

### Statistical analysis

The six independent variables (five motivation scores and one intention score) were treated as continuous variables. Spearman’s correlations were used to explore associations between variables. χ^2^ tests and t-tests were conducted to examine the differences between included and excluded participants. Multivariable linear regression models were used to examine associations between the six independent variables with mean MVPA minutes per day and accelerometer CPM for parents and children (ie, a total of 24 associations: six independent variables with two outcomes in two groups (parent and child). Each model was adjusted for parent sex, number of children in the household and IMD score as these have been previously associated with PA in adults.^6 18 45^ Robust standard errors were used to take account for clustering of children within schools. When designing this paper, we intended to conduct mediation analysis of parent exercise motivation on parent and child MVPA via intention to engage in family-based activity. However, as no associations were found between intentions and either parent or child MVPA, mediation analysis could not be conducted. All analyses were performed in Stata V.12.0.^46^

## Results

### Descriptive characteristics

Descriptive characteristics of the study sample have been reported previously.^12^ Correlation results are presented in table 1. Generally, parents were female (76.1%), with an average two children per household, were autonomously motivated to exercise and expressed intention to engage in regular family-based activity at least three times per week. The mean IMD score fell within the middle quintile, however a greater proportion of families were in the two least deprived quintiles (40.4% and 23.8%), compared with the middle quintile (15.7%) and the two most deprived quintiles (10.9% and 9.3%). The average daily time spent in MVPA exceeded the recommended guidelines for adults (mean (SD); 49.7 (24.8) min) and children (67.6 (20.7) min).^47^ Mean child age was 6.01 (SD: 0.42) years, and mean parent age was 37.75 (SD: 5.68 years). Included participants were generally similar to participants who were excluded due to missing data (table 2). However, excluded participants were more likely to be female (p=0.03), with less children (p=0.02), more deprived (p<0.001), higher amotivation (p<0.001) and external regulation (p=0.04), and lower identified regulation (p<0.001) and intrinsic motivation (p<0.001).

**Table 1:** Means, SD and intercorrelations of the study variables (n=1067)

	Mean	SD	1	2	3	4	5	6	7	8	9	10	11
1. Parent sex (% mother)	*76.1* *%*	*-*	-	-	-	-	-	-	-	-	-	-	-
2. No. of children	2.1	0.9	−0.05 (0.12)	-	-	-	-	-	-	-	-	-	-
3. IMD	15.0	12.8	0.07 (0.03)	−0.10 (0.002)	-	-	-	-	-	-	-	-	-
4. Parent MVPA	49.7	24.8	−0.06 (0.05)	−0.003 (0.94)	0.004 (0.91)	-	-	-	-	-	-	-	-
5. Child MVPA	67.6	20.7	0.06 (0.39)	0.02 (0.49)	−0.04 (0.25)	0.09 (0.005)	-	-	-	-	-	-	-
6. Amotivation	0.24	0.5	0.003 (0.91)	−0.05 (0.13)	0.10 (<0.001)	−0.12 (<0.001)	−0.03 (0.32)	-	-	-	-	-	-
7. External regulation	0.28	0.5	−0.006 (0.85)	0.007 (0.81)	0.02 (0.58)	−0.04 (0.22)	−0.05 (0.09)	0.27 (<0.001)	-	-	-	-	-
8. Introjected regulation	1.26	1.0	0.04 (0.25)	0.004 (0.89)	−0.07 (0.03)	0.11 (<0.001)	0.03 (0.27)	−0.09 (0.006)	0.26 (<0.001)	-	-	-	-
9. Identified regulation	2.64	1.0	−0.07 (0.02)	−0.002 (0.95)	−0.17 (<0.001)	0.23 (<0.001)	0.07 (0.03)	−0.43 (<0.001)	−0.09 (0.005)	0.37 (<0.001)	-	-	-
10. Intrinsic motivation	2.56	1.1	−0.09 (0.004)	0.01 (0.74)	−0.15 (<0.001)	0.20 (<0.001)	0.05 (0.08)	−0.42 (<0.001)	−0.17 (<0.001)	0.23 (<0.001)	0.79 (<0.001)	-	-
11. Intention	3.06	2.0	−0.006 (0.84)	0.0009 (0.98)	0.05 (0.15)	0.08 (0.02)	0.03 (0.35)	−0.05 (0.13)	−0.009 (0.78)	0.06 (0.07)	0.09 (0.005)	0.10 (0.002)	-

Sample sizes ranged from 1014 to 1067. p Values for intercorrelations are reported in parentheses.

IMD, Indices of Multiple Deprivation;  MVPA, moderate-to-vigorous-intensity physical activity.

**Table 2:** Differences between included and excluded participants for each of the study variables

	Included*		Excluded		p
	n	Mean (SD)	n	Mean (SD)	
Sex (% mothers)	911	74.5% (0.44)	325	80.6% (0.40)	0.03
Children in household	911	2.15 (0.94)	130	1.95 (1.00)	0.02
IMD score	911	13.96 (11.93)	257	18.54 (15.09)	<0.001
Amotivation	911	0.20 (0.48)	148	0.46 (0.79)	<0.001
External regulation	911	0.27 (0.47)	145	0.36 (0.67)	0.04
Introjected regulation	911	1.27 (1.01)	151	1.16 (1.07)	0.23
Identified regulation	911	2.69 (0.96)	144	2.32 (1.08)	<0.001
Intrinsic motivation	911	2.61 (1.09)	143	2.23 (1.14)	<0.001
Intentions	911	3.05 (2.03)	107	3.11 (2.07)	0.76
Parent MVPA	911	49.98 (24.42)	251	48.75 (26.10)	0.49
Child MVPA	895	67.65 (20.32)	304	67.36 (21.91)	0.84

*Included participants were parents with complete data for each of the study variables, child MVPA was treated as a separate variable.

IMD, Indices of Multiple Deprivation;  MVPA, moderate-to-vigorous-intensity physical activity.

### Parents’ physical activity

There was strong evidence, in the unadjusted models, that amotivation was negatively associated with parents’ MVPA (*b*: −5.53, 95% CI: −8.30 to −2.75, p<0.001), while introjected regulation (2.53, 1.06 to 3.99, p=0.001), identified regulation (5.76, 4.28 to 7.25, p<0.001) and intrinsic motivation (4.44, 3.10 to 5.78, p<0.001) were positively associated with MVPA (table 3). There was also evidence that parents’ intention to engage in regular family-based activity was positively but weakly associated with MVPA (0.91, 0.16 to 1.67, p=0.02). In the fully adjusted models (R^2^=0.14), every one-unit increase in identified regulation was associated with 6.08 (3.27 to 8.89, p<0.001) min-per day greater MVPA for parents.

**Table 3:** Linear regression analyses showing associations between parent exercise motivation, intention for family activity and their own physical activity

	Unadjusted				Fully adjusted		
Predictor variable	Coeff^*^	95% CI	p		Coeff.	95% CI	p
Personal factors							
Parent sex			0.05				0.15
*Male*		Reference†				Reference	
*Female*	−3.46	−6.90 to −0.03			−2.60	−6.13 to 0.93	
Number of children	−0.08	−1.68 to 1.52	0.92		−0.14	−1.78 to 1.50	0.87
IMD score	−0.001	−0.13 to 0.13	0.98		0.03	−0.11 to 0.16	0.72
Psychosocial factors							
Motivation to exercise							
*Amotivation*	−5.53	−8.30 to −2.75	<0.001		−0.53	−4.16 to 3.11	0.78
*External regulation*	−2.05	−5.08 to 0.99	0.19		−1.52	−5.07 to 2.04	0.40
*Introjected regulation*	2.53	1.06 to 3.99	0.001		0.73	−1.01 to 2.46	0.41
*Identified regulation*	5.76	4.28 to 7.25	<0.001		6.08	3.27 to 8.89	<0.001
*Intrinsic motivation*	4.44	3.10 to 5.78	<0.001		−0.35	−2.65 to 1.95	0.77
Intention to engage in family- based PA	0.91	0.16 to 1.67	0.02		0.67	−0.08 to 1.43	0.08

Sample sizes for the unadjusted analyses ranged from 968 to 1016, and for the adjusted analysis was 911. All analyses adjusted for clustering at the school level.

*Regression coefficients are unstandardised.

†Male sex was used as a reference category, and given a value of zero, against which the effects of the female sex category were assessed.

IMD, Indices of Multiple Deprivation; PA, physical activity.

### Child’s physical activity

For child MVPA, there was evidence in the unadjusted models that both parents’ identified regulation (1.51, 0.28 to 2.74, p=0.02) and intrinsic motivation (1.27, 0.17 to 2.36, p=0.02) were positively associated with MVPA (table 4). In the fully adjusted models (R^2^=0.18), there was weak evidence that parents’ external regulation was associated with a 2.93 (−5.83 to −0.03, p=0.05) min-per-day reduction in children’s MVPA.

**Table 4:** Linear regression analyses showing associations between parent exercise motivation, intention for family activity and their child’s physical activity

	Unadjusted			Fully adjusted		
Predictor variable	Coeff^*^	95% CI	p	Coeff.	95% CI	p
Personal factors						
Parent sex			0.07			0.02
*Male*		Reference†			Reference	
*Female*	2.55	−0.19 to 5.28		3.32	0.47 to 6.16	
Number of children	0.11	−1.22 to 1.44	0.87	0.11	−1.27 to 1.48	0.88
IMD score	−0.05	−0.16 to 0.07	0.45	−0.07	−0.20 to 0.05	0.26
Psychosocial factors						
Motivation to exercise						
*Amotivation*	−0.92	−3.13 to 1.29	0.42	0.34	−2.59 to 3.27	0.82
*External regulation*	−2.16	−4.57 to 0.24	0.08	−2.93	−5.83 to −0.03	0.05
*Introjected regulation*	0.85	−0.33 to 2.04	0.16	0.47	−0.94 to 1.89	0.51
*Identified regulation*	1.51	0.28 to 2.74	0.02	0.97	−1.31 to 3.25	0.40
*Intrinsic motivation*	1.27	0.17 to 2.36	0.02	0.25	−1.63 to 2.12	0.80
Intention to engage in family-based PA	0.34	−0.26 to 0.95	0.27	0.45	−0.17 to 1.07	0.15

*Regression coefficients are unstandardised.

†Male sex was used as a reference category, and given a value of zero, against which the effects of the female sex category were assessed. Sample sizes for the unadjusted analyses ranged from 986 to 1037, and for the adjusted analysis was 926. All analyses adjusted for clustering at the school level.

IMD, Indices of Multiple Deprivation; PA, physical activity.

### Accelerometer CPM

No associations were found between motivation or intention and accelerometer CPM for parents in either the unadjusted or fully adjusted models (table 5). Parents’ external regulation was associated with a 29.3 (−53.8 to −4.7, p=0.02) CPM reduction in children’s PA in the fully adjusted model (R^2^=0.20; table 6).

**Table 5:** Linear regression analyses showing associations between parent exercise motivation, intention for family activity and their own accelerometer CPM

	Unadjusted			Fully adjusted		
Predictor variable	Coeff^*^	95% CI	p	Coeff.	95% CI	p
Personal factors						
Parent sex			0.61			0.61
*Male*		Reference†			Reference	
*Female*	53.3	−148.8 to 255.3		59.5	−165.9 to 284.9	
Number of children	−55.9	−151.3 to 39.6	0.25	−65.6	−169.9 to 38.8	0.22
IMD score	−2.6	−9.9 to 4.7	0.48	−3.0	−11.3 to 5.2	0.47
Psychosocial factors						
Motivation to exercise						
*Amotivation*	−69.3	−233.6 to 95.0	0.41	−78.6	−310.6 to 153.5	0.51
*External regulation*	65.2	−113.5 to 244.0	0.47	100.6	−125.7 to 326.9	0.38
*Introjected regulation*	47.1	−39.5 to 133.7	0.29	32.1	−78.6 to 142.7	0.57
*Identified regulation*	42.9	−46.9 to 132.8	0.35	3.1	−174.8 to 181.0	0.97
*Intrinsic motivation*	36.7	−43.6 to 117.0	0.37	19.3	−127.0 to 165.6	0.80
Intention to engage in family-based PA	−4.9	−50.1 to 40.4	0.83	−5.9	−54.0 to 42.2	0.81

*Regression coefficients are unstandardised.

†Male sex was used as a reference category, and given a value of zero, against which the effects of the female sex category were assessed. Sample sizes for the unadjusted analyses ranged from 968 to 1016, and for the adjusted analysis was 911. All analyses adjusted for clustering at the school level.

CPM, counts per minute; IMD, Indices of Multiple Deprivation; PA, physical activity.

**Table 6:** Linear regression analyses showing associations between parent exercise motivation, intention for family activity and their child’s accelerometer CPM

	Unadjusted			Fully adjusted		
Predictor variable	Coeff^*^	95% CI	p	Coeff.	95% CI	p
Personal factors						
Parent sex			0.02			0.01
*Male*		Reference†			Reference	
* Female*	27.8	4.2 to 51.3		31.6	7.6 to 55.6	
Number of children	4.5	−6.8 to 15.9	0.43	4.5	−7.1 to 16.1	0.45
IMD score	−0.2	−1.2 to 0.8	0.73	−0.6	−1.6 to 0.5	0.31
Psychosocial factors						
Motivation to exercise						
*Amotivation*	−3.9	−22.8 to 15.1	0.69	10.3	−14.4 to 35.1	0.41
*External regulation*	−19.2	−40.1 to 1.6	0.07	−29.3	−53.8 to −4.7	0.02
*Introjected regulation*	10.7	0.4 to 20.9	0.04	11.0	−1.0 to 22.9	0.07
*Identified regulation*	8.1	−2.6 to 18.8	0.14	−4.6	−23.9 to 14.6	0.64
*Intrinsic motivation*	8.1	−1.3 to 17.4	0.09	7.9	−7.9 to 23.8	0.33
Intention to engage in family-based PA	4.1	−1.2 to 9.3	0.13	4.9	−0.4 to 10.1	0.07

* Regression coefficients are unstandardised.

†Male sex was used as a reference category, and given a value of zero, against which the effects of the female sex category were assessed. Sample sizes for the unadjusted analyses ranged from 986 to 1037, and for the adjusted analysis was 926. All analyses adjusted for clustering at the school level.

CPM, counts per minute; IMD, Indices of Multiple Deprivation; PA, physical activity.

## Discussion

The data presented indicate that each unit increase in identified regulation was associated with six more minutes per day of MVPA for parents, whereas each unit increase in parental external regulation was weakly associated with almost 3 min per day less child MVPA. Although a 6-min per-day increase in MVPA for adults may seem small, if repeated daily across the week this would equate to an extra 42 min of MVPA, which is almost a third of the recommended guidelines. The 3-min per-day reduction in child MVPA is less compelling, because it accounts for only 5% of their daily recommendation.

Previous research has shown that identified regulation is the dominant motivational factor associated with PA behaviour.^21 48^ Therefore, our finding among parents of young children concurs with research in the general adult population. Identified regulation is motivation rooted in the personal value placed on a behaviour, therefore, logically when a behaviour is accepted as personally important, the behaviour may be prioritised alongside other pressures on parents’ time (eg, childcare). Intrinsic motivation was not associated with parent MVPA in the fully adjusted model, despite proposals in SDT that intrinsic motivation promotes the most positive motivational effects.^20^ One explanation suggests that if the target behaviour (eg, exercise) is not inherently interesting, identified regulation may be a more salient predictor of behaviour than intrinsic motivation,^49 50^ which has been supported by several studies.^21 48^ Participation in such activities is unlikely to be intrinsically driven for most individuals, but rather by what can be obtained from it (eg, health/fitness gains, losing weight).^51^ Even if parents of young children find PA enjoyable, this enjoyment may not be sufficient for parents to be active amid other parenting duties.

Parents’ external regulation was weakly negatively associated with their child’s MVPA, and not associated with their own MVPA. Similarly, introjected regulation was not associated with either child or adult MVPA. These findings suggest that family-based PA interventions which rely on strategies that promote parents’ controlled motivation (eg, offering incentives, motivating through demand compliance or using guilt inducement) may be a poor investment of time and resources as they are unlikely to increase parents’ MVPA, and may have a negative effect on child MVPA. Amotivation was not associated with MVPA for parents or children, consistent with previous literature.^21^ Empirically, it is difficult to distinguish amotivation from a lack of controlled or autonomous regulation.^52^ Additionally, it has been hypothesised that individuals could be autonomously motivated not to exercise, even while perceiving some value in the behaviour.^53 54^ Therefore, amotivation may be confounding the associations between the other regulations in the multivariate analyses.^21^

As a measure of PA volume, accelerometer CPM were not associated with any motivation regulations for parents. One suggestion for this null finding is that CPM takes into account all incidental activity in the sedentary and light domains as well as more vigorous activity, whereas motivations are more likely to be associated with more conscious forms of activity like those accounted for by MVPA thresholds. However, parents’ external regulation was negatively associated with children’s accelerometer CPM, in agreement with the child MVPA data.

Reviews have highlighted that exercise intention is a consistent positive PA correlate in adults.^4 18^ However, other studies have found no association between intention and PA,^55^ or found that medium-sized changes in intention resulted in only trivial changes in behaviour (r=0.06).^56^ In the present study, intention to engage in family-based activity, rather than personal intention, was examined, but no association with MVPA was found. Although scores ranged from 0 to 7, over 60% of parents intended to engage in regular family-based activity either twice or three times weekly, therefore, the lack of variation in intention may partially explain the absence of an association with MVPA. Although family-based activity intentions were measured, PA was measured separately for parents and children, meaning that no information was available on the quantity or quality of MVPA that was completed together as a family.

### Recommendations for future research

Future research with families could combine accelerometry with activity diaries and/or global positioning system (GPS) data,^57 58^ in order to better understand the association between intention to engage in family-based activity if and when family PA occurs. To understand more about how parents are motivated, future research examining parents’ motivations both for their own exercise and their children’s activity levels is warranted. For parents who are active with their children, it would be fruitful to discover whether they are active together because they value the time, believe it is beneficial, feel that they should, or because they have no choice due to issues with childcare logistics or costs. Other suggestions include improving the intention measure by including different contexts (eg, weekday/weekend day, active transportation), and examining links between motivation and other factors that might underpin family PA.

### Strengths and limitations

Strengths of this study included the large sample size with parent–child dyads and the objective measure of PA. However, the use of accelerometry limited our understanding of the types of PA in which parents and children engaged, and whether they engaged together or separately. The cross-sectional design of the study prevented the direction of the associations observed being assessed. The majority of parents met the recommended PA levels, suggesting this sample was not representative of the wider parent population, limiting the generalisability of the findings. PA levels may have been influenced by parents being informed that the study was measuring PA behaviour, and thus more active parents may have chosen to participate, resulting in self-selection bias. Additionally, ‘wear effect’ may have been a factor, where participants were more active than usual due to them being involved in the study and wearing an accelerometer, particularly in the first day of data collection.^59^ Participants included in the final analysis were generally similar to those excluded, however, minor differences were present in their reported motivation scores, deprivation, number of children and parent's sex, which may have led to missing data bias. Additionally, a total of 24 associations were tested, therefore it is possible that associations were affected by chance.

## Conclusion

This study is the first to examine parents’ autonomous and controlled motivation to exercise and intention to engage in family-based activity in relation to both parent and child objectively measured MVPA. The results demonstrate that despite the challenges and time constraints of being a parent of a young child, the associations between motivation regulations and PA are similar in pattern to findings from the general adult population. Parents’ motivation which stemmed from a personal valuing of exercise was associated with greater parent MVPA, whereas motivation based on pressure and coercion was negatively associated with their child’s CPM and MVPA, although this association was weak, and so may be due to chance. Intention to engage in family-based PA was not associated with parent or child MVPA. These results highlight the potential benefits of focusing future family-based PA interventions on helping parents identify with personally meaningful or valuable benefits of exercise while avoiding the use of external contingencies to motivate behaviour.
